# Acupuncture and moxibustion therapy for scapulohumeral periarthritis: Protocol for an overview of systematic reviews and meta-analysis

**DOI:** 10.1097/MD.0000000000021567

**Published:** 2020-08-28

**Authors:** Zenan Wu, Xinyu Yu, Jun Xiong, Guoxin Wu, Zhengyun Zuo, Qiongshan Xie

**Affiliations:** a Jiangxi University of Traditional Chinese Medicine; b The Affiliated Hospital of Jiangxi University of Traditional Chinese Medicine, Nanchang; c Chengdu University of Traditional Chinese Medicine, Chengdu, China.

**Keywords:** acupuncture, moxibustion, scapulohumeral periarthritis, protocol, overview

## Abstract

**Background:**

Scapulohumeral periarthritis (SP) is a very common painful shoulder disorder. Several systematic reviews (SRs) and meta-analyses have reported the effectiveness of acupuncture for patients with SP. However, the evidence has not been systematically synthesized. This overview aims to map, synthesize, and assess the reliability of evidence generated from these SRs and meta-analyses of acupuncture for SP.

**Methods:**

We will electronically search the following databases for literature, regardless of publication status and language: the Cochrane Central Register of Controlled Trials (CENTRAL); PubMed; EMBASE; China National Knowledge Infrastructure (CNKI); Chinese Biomedical Literature Database (CBM); Chinese Scientific Journal Database (VIPdatabase); and Wan-Fang Database. In order to ensure the comprehensiveness and accuracy of the literature retrieval, we will combine the Suggestions of evidence-based medicine experts with the actual situation in the literature retrieval process to formulate the retrieval strategy, and make corresponding records to find the most appropriate retrieval strategy. The reference lists and the citation lists of studies meeting the inclusion criteria and relevant SRs will also be searched to identify further studies for inclusion. Before this review completed, the two reviewers will conduct the searching once again to ensure the latest studies could be included.

**Ethics and dissemination:**

Ethical approval is not required for overviews. We plan to publish results in peer-reviewed journals and present at international and national academic, clinical, and patient conferences.

**Results:**

The results will be published in a peer-reviewed journal.

**Conclusion:**

This overview will provide comprehensive evidence of acupuncture for patients with SP.

**INPLASY registration number:**

INPLASY202060020.

## 1 Introduction

### 1.1 Description of the condition

Scapulohumeral periarthritis (SP), as known as frozen shoulder or adhesive periarthritis of shoulder, is a common musculoskeletal disorder in middle-aged people. It commonly displays shoulder ache and shoulder dyskinesia as characteristic caused by intraarticular and extraarticular adhesion gradually, which is a chronic inflammation and degenerative disease located in soft tissue, such as shoulder muscles, tendons, ligaments, and capsule.^[1–3]^ The incidence of SP is estimated to be between 3% and 5% of the population.^[4,5]^ In china, at around 8% of adults have had scapulohumeral periarthritis, between 40 and 60 years old predominantly especially female more than male and the SP can have serious influences on patients’ health and quality of life. Shoulder pain and limited mobility affect people in every way, prompting treatment for this disease is essential.^[6,7]^

The treatments of SP are mainly conservative, including intra-articular triamcinolone injection and bupivacaine suprascapular nerve blocks.^[4,8]^ Numerous studies have demonstrated the efficacy of intra-articular (IA) steroid injection in pain reduction, shoulder function, and range of motion (ROM) improvement in the short term. Moreover, these treatment options may cause severe adverse events, such as infectious arthritis and cartilage damage.^[9,10]^

Acupuncture can relieve pain, improve the blood circulation, stimulate metabolism of local tissue, etc^[11]^ acupuncture is an extensively accepted alternative therapy, as an important part of complementary and complementary medicine, has gained increased popularity for the management of constipation around the world.^[12]^ acupuncture has the functions of enhancing immunity, regulating blood circulation, and preventing diseases. Some clinical trials have found that acupuncture has a significant impact on SP.^[13]^ In addition, it has the advantages of safe, reliable, and is easy to use without toxic and side effects. However, the evidence has not been systematically synthesized. This overview aims to map, synthesize, and assess the reliability of evidence generated from these systematic reviews (SRs) and meta-analyses of acupuncture for SP. Thus, this study will investigate the effect and safety of acupuncture and moxibustion for patients with SP.

### 1.2 Description of the intervention

Acupuncture is an ancient treatment method based on traditional Chinese medicine (TCM), which has existed in China for about 2500 years. It mainly uses acupuncture to stimulate specific acupoints or surface parts of the body to stimulate the related functions of acupoints, and stimulates the meridians and vital energy to regulate the zang-fu organs, so as to treat diseases.^[14]^ It is believed that acupuncture works by releasing endogenous opioids in the body that relieve pain, by overriding pain signals in the nerves, or by allowing energy (qi) or blood to flow freely through the body.^[15]^ Some studies have shown that acupuncture therapy of acute periarthritis of scapulohumeral suggests filiform acupuncture, distal acupoint selection and strong stimulation of purging. Among them, two schemes of “Tiaokou point penetrating Chengshan Point” and “local adjacent point cooperating with Tiaokou point” are recommended for needle acupuncture.^[16,17]^

Moxibustion has a significant effect on analgesia of SP, with less trauma, less risk, and less adverse reactions.^[18]^ Nowadays, the indications for moxibustion are gradually expanded because of its function and practicality, The heat generated by moxibustion can effectively play the role of replenishing Yang and dispelling cold, promoting blood circulation and removing blood stasis, and warm the meridians and collaterals.^[19]^ It has been found in modern studies that the warm effect and light radiation produced by moxibustion are one of the most important factors in the effective treatment of SP. The heat of combustion can promote blood circulation, relax blood vessels, and regulate the body's immune system. The far infrared ray produced by moxibustion can directly act on the superficial part of the human body and diffuse the heat by conduction. The near infrared ray energy is strong, can penetrate into the deep tissue directly, provides the necessary energy for the body cell activity.^[20]^

### 1.3 Description of the objectives

The objectives of this study are as follows:

1. Explore the methodological and reporting quality of available SRs using the Assessment of Multiple systematic Reviews-2 (AMSTAR-2)^[21]^ and Preferred Reporting Items for Systematic Reviews and Meta-analyses (PRISMA)^[22]^
2. Summarize the best current evidence for the effectiveness and safety of acupuncture and moxibustion for SP through qualitative analysis of the outcomes.
3. Provide more reliable, evidence-based medical references for clinical practitioners and researchers

## 2 Methods

### 2.1 Study registration

It is an overview protocol that follows the recommendations of the Cochrane Handbook for Systematic Reviews of Interventions.^[23]^ This protocol was recorded in the International Platform of Registered Systematic Review and Meta-analysis Protocols (INPLASY), registration number INPLASY202060020 (https://inplasy.com/inplasy-2020-6-0020/). And if there are any changes, we will describe it in our full review.

### 2.2 Inclusion and exclusion criteria

Population, Intervention, Comparison, Outcome and Study (PICOS) strategy was employed.

#### 2.2.1 Types of study

For this overview, only SRs and meta-analysis of randomized controlled trials (RCTs) for moxibustion in people with Scapulohumeral periarthritis, published in English and Chinese, will be included. All relevant SRs and meta-analyses of randomized clinical trials published in English and Chinese about acupuncture for SP will be included.

#### 2.2.2 Type of participants

Study participants of different age ranges with all types of SP will be included, regardless of sex, race, occupation, education, nationality, etiology, and severity.

#### 2.2.3 Type of interventions

Acupuncture (it includes body acupuncture, electroacupuncture, fire acupuncture, acupoint injection, and ear acupuncture) and moxibustion (it includes suspended moxibustion, Thunder fire moxibustion, taiyi miraculous moxa roll, mild moxibustion, needle warming moxibustion) will be included as a single intervention or major part of a combination therapy with other active interventions (e.g., western medicine, or cupping).

#### 2.2.4 Type of comparator(s)/control

The comparative interventions will be sham acupuncture, sham moxibustion, placebo, no treatment, or other active treatments. The following treatment comparisons will be performed:

1. Acupuncture and moxibustion vs no treatment
2. Acupuncture and moxibustion vs placebo or sham acupuncture or sham moxibustion
3. Acupuncture and moxibustion vs other active therapies
4. Acupuncture and moxibustion + active therapy vs the same active therapy

#### 2.2.5 Types of outcome measurements

##### 2.2.5.1 Main outcomes

Main outcome indicators: pain relief visual analog scale (VAS) score, ROM, Melle Score of shoulder joint functional activity or other validated scales, at least after one week of treatment.

##### 2.2.5.2 Additional outcomes

Additional outcomes include the following aspects:

1. Secondary outcome indicators: total effective rate;
2. Safety outcome: adverse reactions.

#### 2.2.6 Study design

### 2.3 Search methods for identification of studies

We will electronically search the following databases for literature, regardless of publication status and language: the Cochrane Central Register of Controlled Trials (CENTRAL); PubMed; EMBASE; China National Knowledge Infrastructure (CNKI); Chinese Biomedical Literature Database (CBM); Chinese Scientific Journal Database (VIP database); and Wan-Fang Database. In order to ensure the comprehensiveness and accuracy of the literature retrieval, we will combine the Suggestions of evidence-based medicine experts with the actual situation in the literature retrieval process to formulate the retrieval strategy, and make corresponding records to find the most appropriate retrieval strategy. The reference lists and the citation lists of studies meeting the inclusion criteria and relevant SRs will also be searched to identify further studies for inclusion. Before this review completed, the two reviewers will conduct the searching once again to ensure the latest studies could be included.

The search will be restricted to human subjects, while there is no restriction on any specific languages. The proposed search strategy for PubMed is presented in Table 1.

**Table 1:**
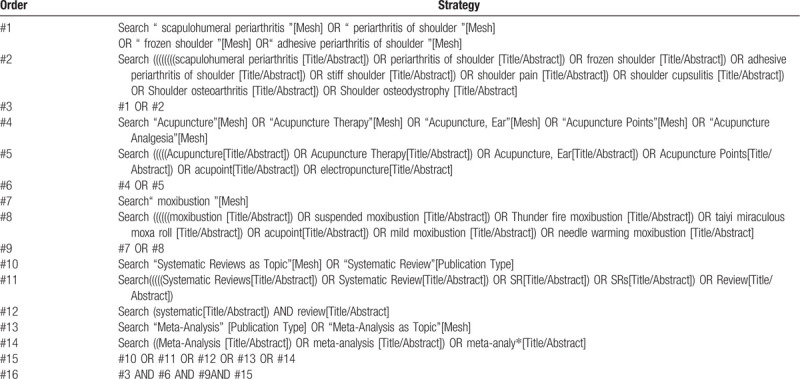
Search strategy (PubMed).

### 2.4 Studies selection

**Figure 1:**
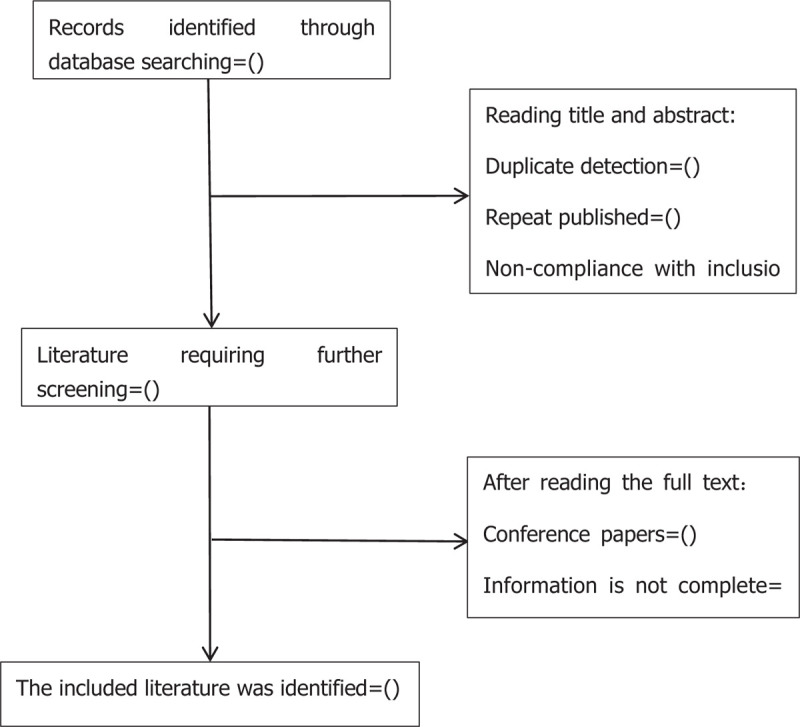
Flowchart of literature selection.

Studies will be identified using NoteExpress 3.0. After the initial removal of duplicate studies, two reviewers (ZNW and YXY) will independently screen titles and abstracts based on the eligibility criteria. Full-text studies will be retrieved for all potentially includable SRs or SR protocols. If studies contain insufficient information to make a decision about eligibility, QSX will try to contact authors of the original reports to obtain further details. During the procedure, disagreements will be resolved by discussion or consensus with the third reviewer (GXW). Study selection will be performed in accordance with the PRISMA flowchart (Fig. 1).

### 2.5 Data extraction

Two researchers (ZNW and YXY) extracted the literature information according to the inclusion and exclusion criteria, including the following aspects:

1. Study characteristics: author, year, study design, sample size and follow-up time;
2. Patient characteristics: age, sex, and type of SP;
3. Intervention: intervention measures in the experimental group, intervention measures in the control group);
4. Outcome of the study: Two researchers (GXW and QSX) cross-examined the results of extraction of the included literature. In case of differences, a third party (JX) should be consulted to resolve them.

### 2.6 Evaluation of the methodological and reporting quality of the included studies

For each SRs that meets the inclusion criteria, two reviewers (ZNW and YXY) will use the Assessment of Multiple systematic Reviews-2 (AMSTAR-2) Measurement Tool and PRISMA to assess methodological quality and report quality. At the same time, differences arising during the evaluation process will be discussed by two reviewers and arbitrated by a third author (JX).

1. AMSTAR-2 consists of 16 items, 7 of which are critical areas. The 16 criteria are rated as “yes” (clearly completed), “no” (clearly not completed), “cannot report” (unclear completion), or “not applicable” based on the information provided by the reviewer to the system review when the criteria are met.
2. PRISMA: This is a 27-item list. Each checklist item will be evaluated as yes, no, or partially Yes to indicate compliance.

### 2.7 Evaluation of the evidence quality of the included studies

After the evidence is evaluated by the Grading of Recommendations Assessment, Development and Evaluation (GRADE), the quality of the evidence, the weights of strengths and weaknesses, and the patient's values and preferences will be carefully considered by two authors (ZNW and YXY) to develop preliminary recommendations. This proposal will be reviewed by 2 to 3 rounds of Delphi process,^[24]^ which will then be submitted to other two authors (GXW and ZYZ) for approval. We will use the GRADE Grid instrument^[25]^ to review each recommendation one by one to group it into one of five options, including “strong recommendation,” “weak recommendation,” “unclear recommendation,” “weak no recommendation,” and “strong no recommendation.” The aim is to reach a better consensus. If 75% of the experts agree on an option, there is consensus on the recommendation. Otherwise, the project goes to the next Delphi process to discuss the disputed project again.

### 2.8 Dealing with lost data

When we find insufficient or unspecified data in the published SRs, it will be necessary to contact the author by phone or email with the necessary information. If the data we need cannot be collected completely, it will be considered useless and abandoned. After that, we will analyze the existing data and discuss the potential impact of missing data

### 2.9 Synthesis of data

Two of our researchers (QSX and ZNW) will use the bias risk tool provided by the Cochrane Collaboration to evaluate the quality of the literature using RevMan 5.3 software. This recommended tool includes 7 important items: sequence generation, allocation concealment, blinding of participants and personnel, blinding of results evaluation, incomplete result data, selective result reporting, and other biases. Make “Low risk,” “High risk,” and “unclear risk” judgments for each research literature. Finally, a “risk of deviation” summary and a chart are generated to show the results. As with the previous process, it will be independently assessed by 2 researchers. If there is disagreement, it will be discussed with the 3rd researcher (JX).

RevMan 5.3.3 software was used for Meta analysis of the research objects. Counting data were represented by relative risk (RR) and its 95%CI, and measurement data were represented by mean difference (MD) and its 95%CI. Heterogeneity among all included studies was detected by χ^2^ test. When statistical homogeneity was found among the results (*P* > .1, *I*^2^ < 50%), a fixed effect model was used for meta-analysis of the results. If statistical heterogeneity exists among the results (*P* < .1, *I*^2^ > 50%), the source of heterogeneity needs to be analyzed. If statistical heterogeneity exists between the two results and the difference is not statistically significant, a random-effects model should be used for Meta analysis. When there is too much heterogeneity among the results of various studies, statistical methods such as subgroup analysis, sensitivity analysis, and descriptive analysis can be used to treat the heterogeneity.

## 3 Discussion

We have developed this study based on the principles and standards of evidence-based medicine, in collaboration with multidisciplinary experts, which will facilitate the treatment of SP by clinicians, as well as for teaching and educating patients.

Based on what we know so far, we have found some limitations of this Protocol:

1. Most of the sites included in the study are in China;
2. The application of acupuncture and moxibustion in other countries and regions needs further study.
3. At the same time, the differences of acupuncture and moxibustion operation methods between different countries should also be need considered.

This study has a number of contributions:

1. to the best of our knowledge, this is the first protocol to assess acupuncture and moxibustion therapy for patients with SP;
2. the results of this protocol will be beneficial to acupuncturists and physicians to make decisions the optimal method of treating the disease, and help patients with SP seeking optimal treatment;
3. the results are helpful to find out the correct operation method of acupuncture and moxibustion for treating SP and the relationship between the therapeutic effect, the time of acupuncture and moxibustion and the total amount of acupuncture and moxibustion, effectively improving the efficacy and safety of SP with acupuncture and moxibustion.

## Author contributions

**Conceptualization:** GuoXin Wu, Jun Xiong, ZhengYun Zuo.

**Data curation:** GuoXin Wu, XingYu Yu, ZeNanWu.

**Formal analysis:** XingYu Yu, QingShan Xie.

**Investigation:** Jun Xiong, GuoXin Wu. Methodology: Jun Xiong, XingYu Yu, ZeNan Wu.

**Software:** GuoXin Wu, QingShan Xie.

**Supervision:** Jun Xiong, ZhengYun Zuo.

**Writing – original draft:** Jun Xiong, GuoXin Wu, XingYu Yu, ZeNan Wu.
